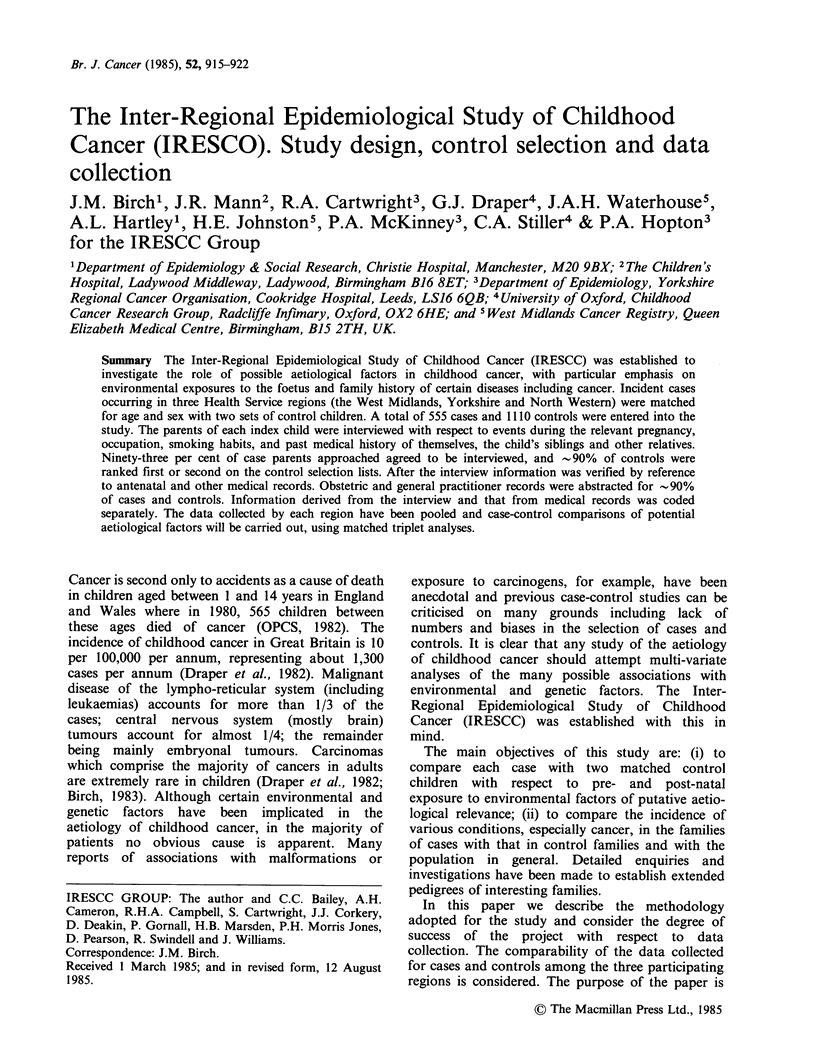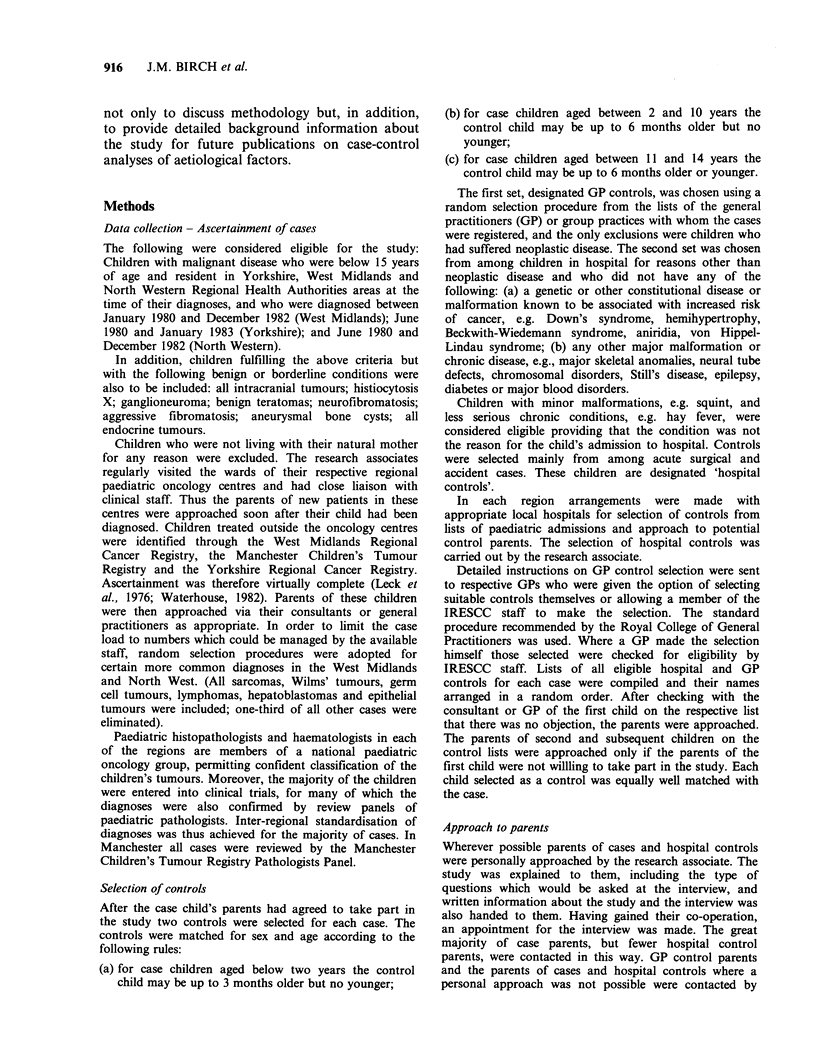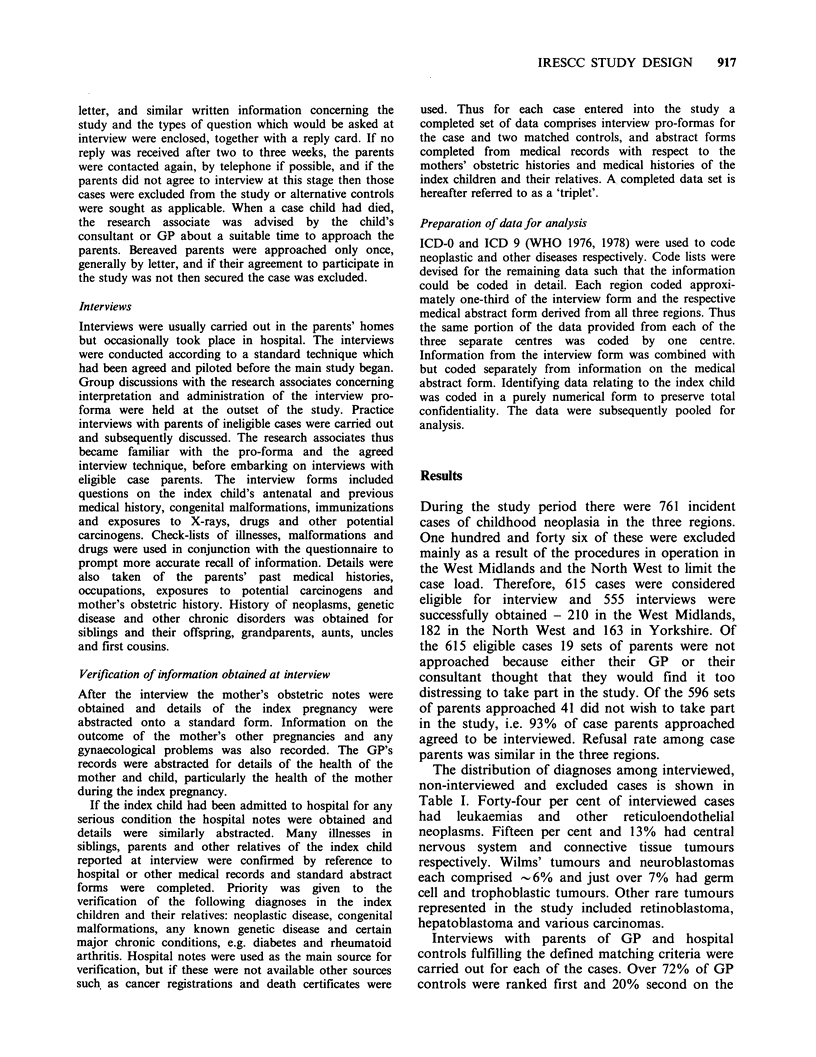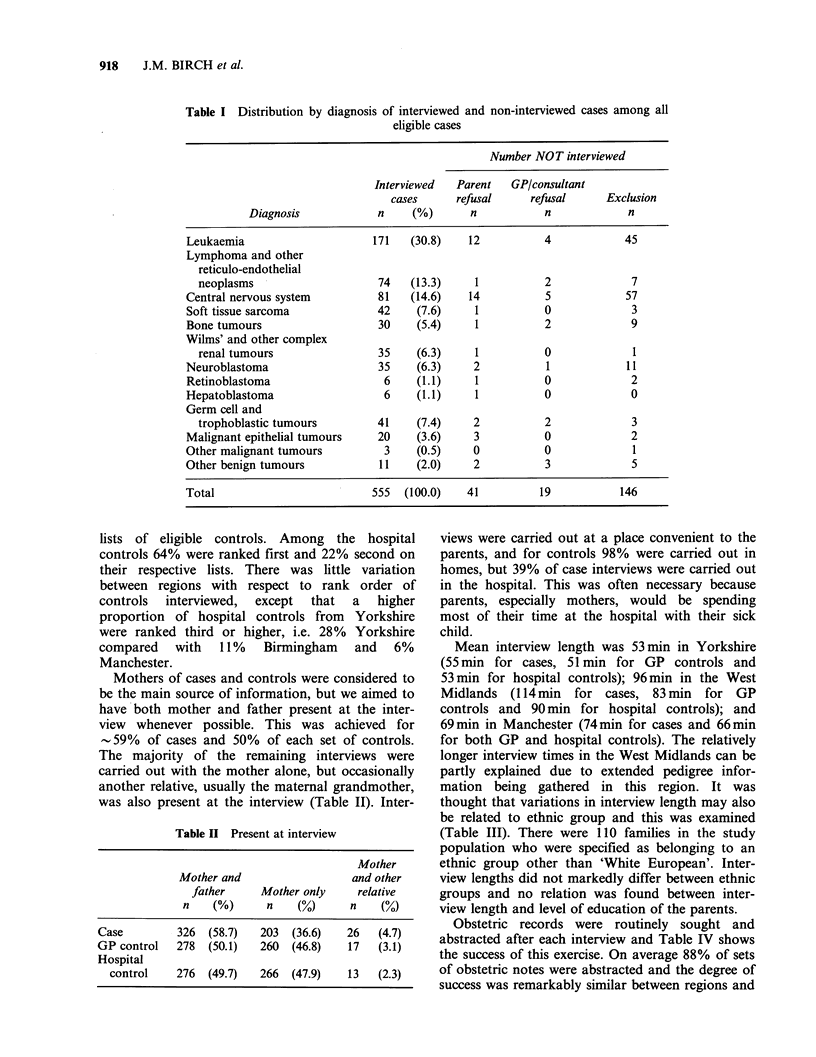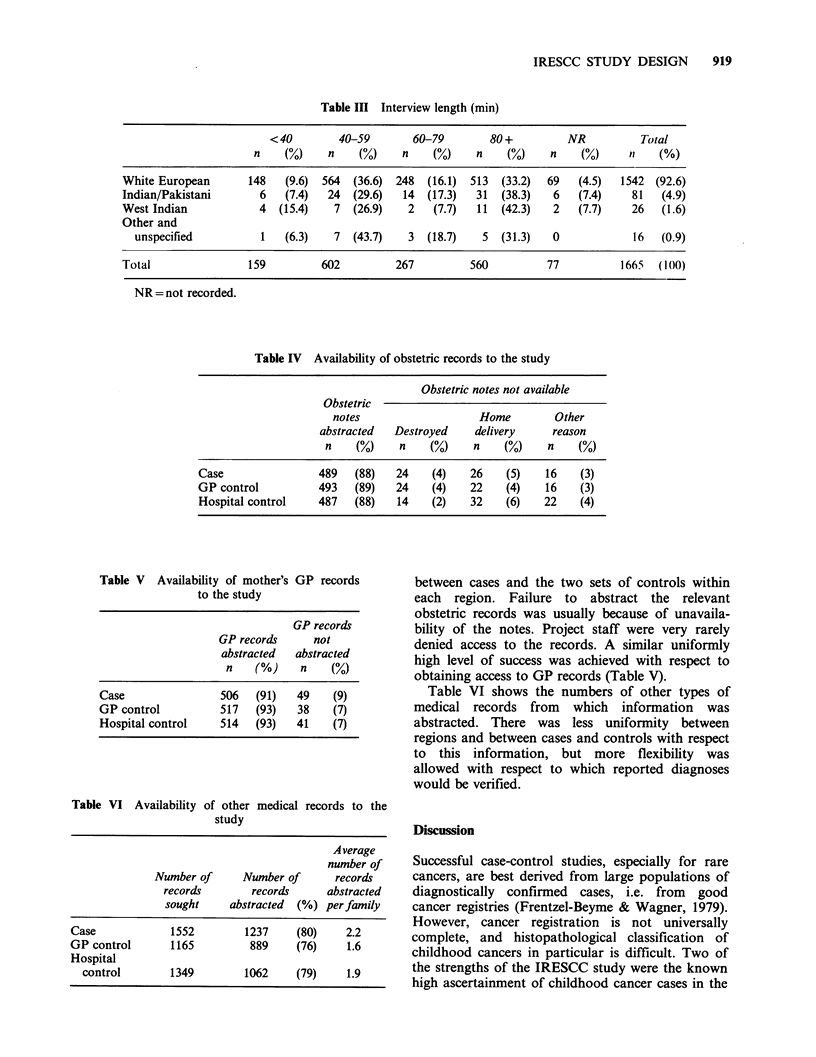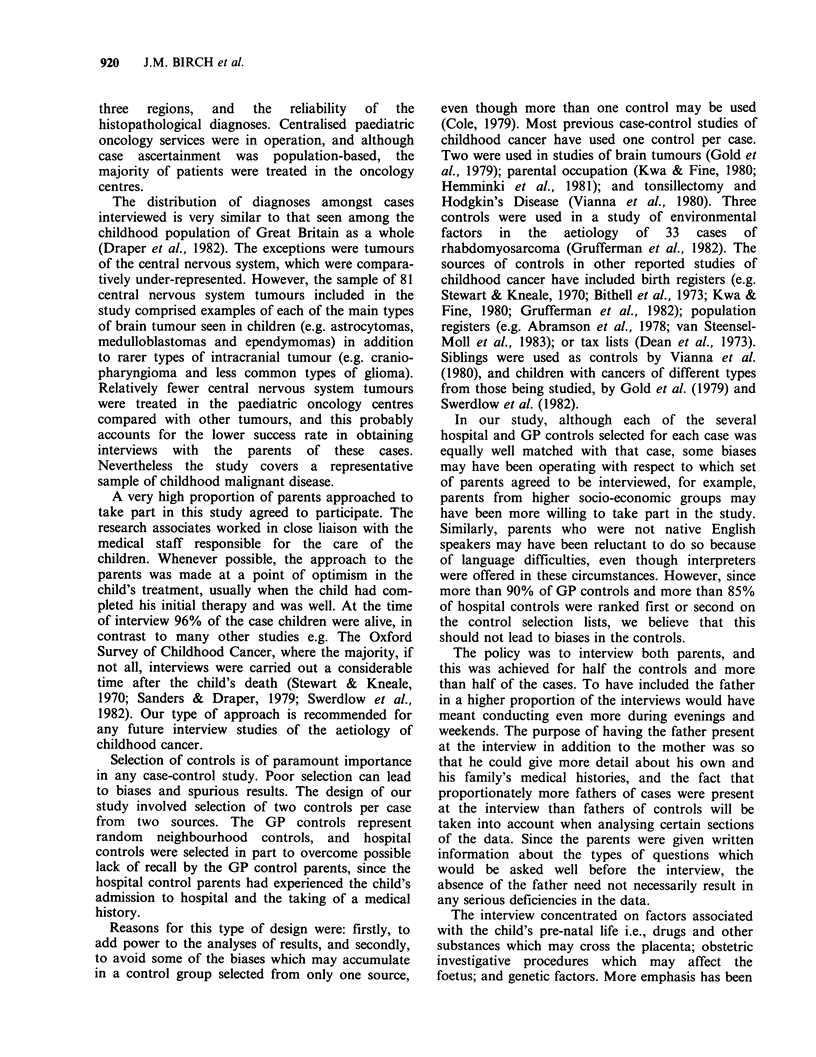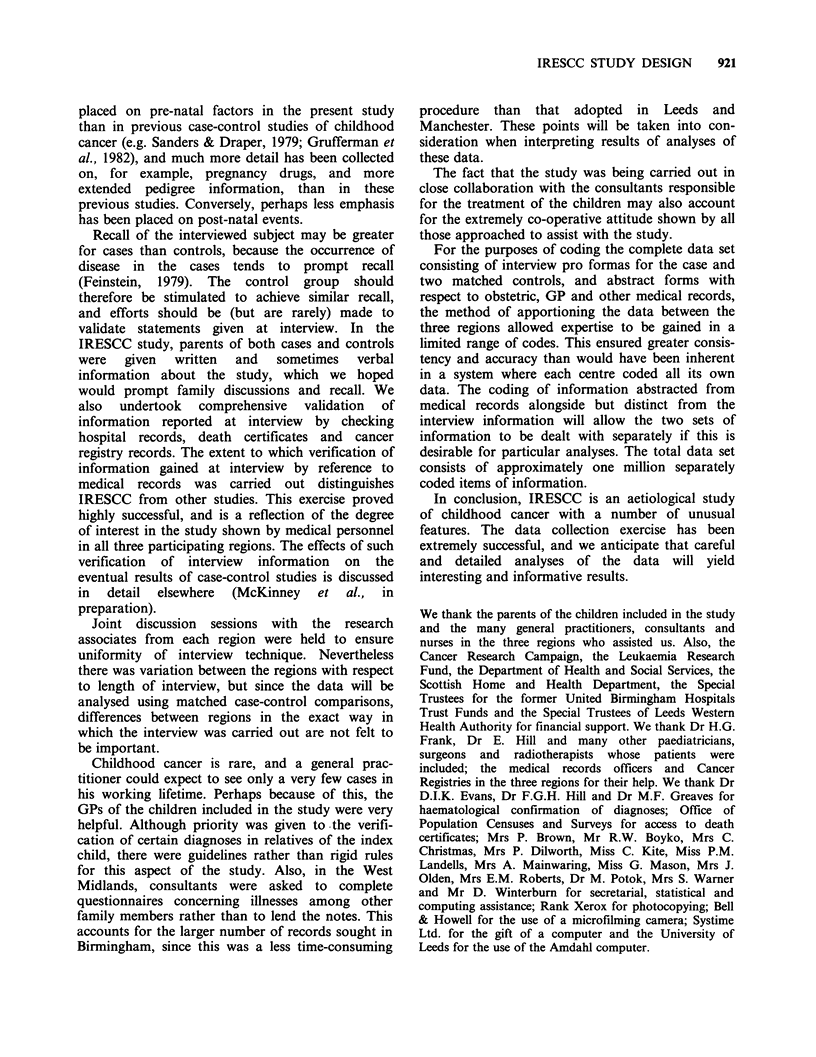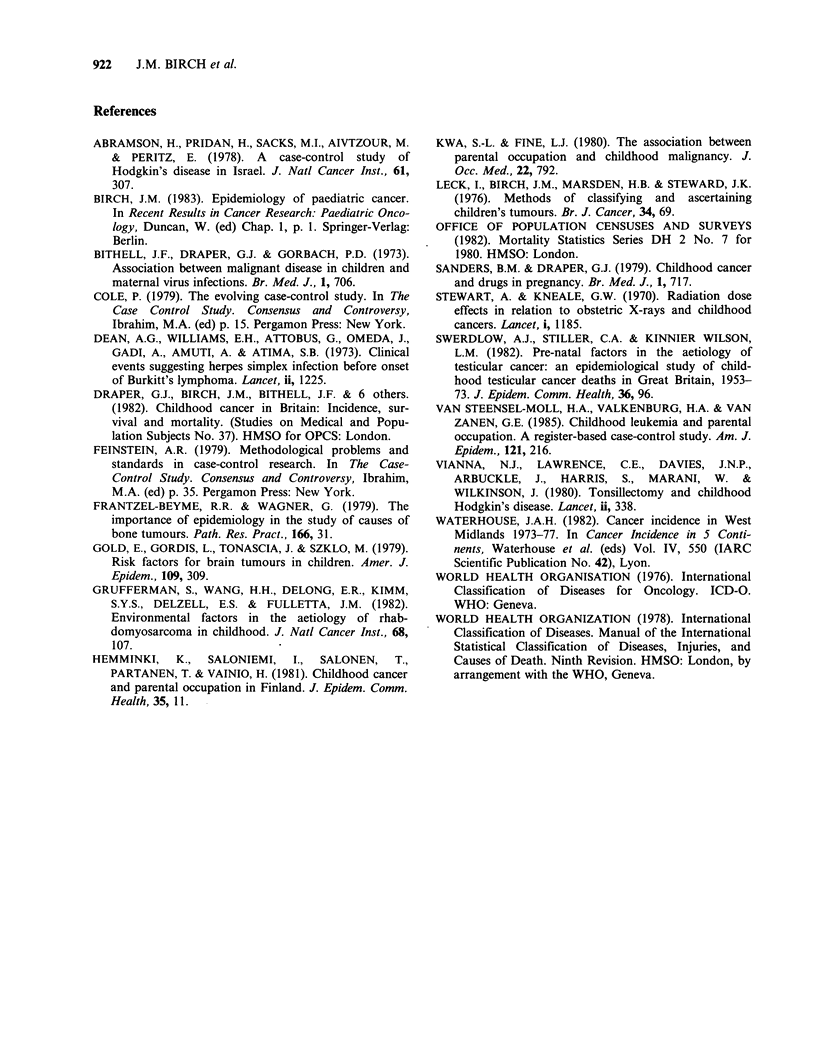# The Inter-Regional Epidemiological Study of Childhood Cancer (IRESCO). Study design, control selection and data collection.

**DOI:** 10.1038/bjc.1985.277

**Published:** 1985-12

**Authors:** J. M. Birch, J. R. Mann, R. A. Cartwright, G. J. Draper, J. A. Waterhouse, A. L. Hartley, H. E. Johnston, P. A. McKinney, C. A. Stiller, P. A. Hopton

## Abstract

The Inter-Regional Epidemiological Study of Childhood Cancer (IRESCC) was established to investigate the role of possible aetiological factors in childhood cancer, with particular emphasis on environmental exposures to the foetus and family history of certain diseases including cancer. Incident cases occurring in three Health Service regions (the West Midlands, Yorkshire and North Western) were matched for age and sex with two sets of control children. A total of 555 cases and 1110 controls were entered into the study. The parents of each index child were interviewed with respect to events during the relevant pregnancy, occupation, smoking habits, and past medical history of themselves, the child's siblings and other relatives. Ninety-three per cent of case parents approached agreed to be interviewed, and approximately 90% of controls were ranked first or second on the control selection lists. After the interview information was verified by reference to antenatal and other medical records. Obstetric and general practitioner records were abstracted for approximately 90% of cases and controls. Information derived from the interview and that from medical records was coded separately. The data collected by each region have been pooled and case-control comparisons of potential aetiological factors will be carried out, using matched triplet analyses.


*Br. J. Cancer* (1985), **52**, 915–922

### The Inter-Regional Epidemiological Study of Childhood Cancer (IRESCO). Study design, control selection and data collection

J.M. Birch[1], J.R. Mann[2], R.A. Cartwright[3], G.J. Draper[4], J.A.H. Waterhouse[5], A.L. Hartley[1], H.E. Johnston[5], P.A. McKinney[3], C.A. Stiller[4] & P.A. Hopton[3] for the IRESCC Group

[1]*Department of Epidemiology & Social Research, Christie Hospital, Manchester, M20 9BX;* [2]*The Children's Hospital, Ladywood Middleway, Ladywood, Birmingham B16 8ET;* [3]*Department of Epidemiology, Yorkshire Regional Cancer Organisation, Cookridge Hospital, Leeds, LS16 6QB;* [4]*University of Oxford, Childhood Cancer Research Group, Radcliffe Infimary, Oxford, OX2 6HE; and* [5]*West Midlands Cancer Registry, Queen Elizabeth Medical Centre, Birmingham, B15 2TH, UK.*

**Summary** The Inter-Regional Epidemiological Study of Childhood Cancer (IRESCC) was established to investigate the role of possible aetiological factors in childhood cancer, with particular emphasis on environmental exposures to the foetus and family history of certain diseases including cancer. Incident cases occurring in three Health Service regions (the West Midlands, Yorkshire and North Western) were matched for age and sex with two sets of control children. A total of 555 cases and 1110 controls were entered into the study. The parents of each index child were interviewed with respect to events during the relevant pregnancy, occupation, smoking habits, and past medical history of themselves, the child's siblings and other relatives. Ninety-three per cent of case parents approached agreed to be interviewed, and ~90% of controls were ranked first or second on the control selection lists. After the interview information was verified by reference to antenatal and other medical records. Obstetric and general practitioner records were abstracted for ~90% of cases and controls. Information derived from the interview and that from medical records was coded separately. The data collected by each region have been pooled and case-control comparisons of potential aetiological factors will be carried out, using matched triplet analyses.

Cancer is second only to accidents as a cause of death in children aged between 1 and 14 years in England and Wales where in 1980, 565 children between these ages died of cancer (OPCS, 1982). The incidence of childhood cancer in Great Britain is 10 per 100,000 per annum, representing about 1,300 cases per annum (Draper *et al.*, 1982). Malignant disease of the lympho-reticular system (including leukaemias) accounts for more than 1/3 of the cases; central nervous system (mostly brain) tumours account for almost 1/4; the remainder being mainly embryonal tumours. Carcinomas which comprise the majority of cancers in adults are extremely rare in children (Draper *et al.*, 1982; Birch, 1983). Although certain environmental and genetic factors have been implicated in the aetiology of childhood cancer, in the majority of patients no obvious cause is apparent. Many reports of associations with malformations or exposure to carcinogens, for example, have been anecdotal and previous case-control studies can be criticised on many grounds including lack of numbers and biases in the selection of cases and controls. It is clear that any study of the aetiology of childhood cancer should attempt multi-variate analyses of the many possible associations with environmental and genetic factors. The Inter-Regional Epidemiological Study of Childhood Cancer (IRESCC) was established with this in mind.

The main objectives of this study are: (i) to compare each case with two matched control children with respect to pre- and post-natal exposure to environmental factors of putative aetiological relevance; (ii) to compare the incidence of various conditions, especially cancer, in the families of cases with that in control families and with the population in general. Detailed enquiries and investigations have been made to establish extended pedigrees of interesting families.

In this paper we describe the methodology adopted for the study and consider the degree of success of the project with respect to data collection. The comparability of the data collected for cases and controls among the three participating regions is considered. The purpose of the paper is

IRESCC GROUP: The author and C.C. Bailey, A.H. Cameron, R.H.A. Campbell, S. Cartwright, J.J. Corkery, D. Deakin, P. Gornall, H.B. Marsden, P.H. Morris Jones, D. Pearson, R. Swindell and J. Williams.

Correspondence: J.M. Birch.

Received 1 March 1985; and in revised form, 12 August 1985.

© The Macmillan Press Ltd., 1985

not only to discuss methodology but, in addition, to provide detailed background information about the study for future publications on case-control analyses of aetiological factors.

### Methods

*Data collection – Ascertainment of cases*

The following were considered eligible for the study: Children with malignant disease who were below 15 years of age and resident in Yorkshire, West Midlands and North Western Regional Health Authorities areas at the time of their diagnoses, and who were diagnosed between January 1980 and December 1982 (West Midlands); June 1980 and January 1983 (Yorkshire); and June 1980 and December 1982 (North Western).

In addition, children fulfilling the above criteria but with the following benign or borderline conditions were also to be included: all intracranial tumours; histiocytosis X; ganglioneuroma; benign teratomas; neurofibromatosis; aggressive fibromatosis; aneurysmal bone cysts; all endocrine tumours.

Children who were not living with their natural mother for any reason were excluded. The research associates regularly visited the wards of their respective regional paediatric oncology centres and had close liaison with clinical staff. Thus the parents of new patients in these centres were approached soon after their child had been diagnosed. Children treated outside the oncology centres were identified through the West Midlands Regional Cancer Registry, the Manchester Children's Tumour Registry and the Yorkshire Regional Cancer Registry. Ascertainment was therefore virtually complete (Leck *et al.*, 1976; Waterhouse, 1982). Parents of these children were then approached via their consultants or general practitioners as appropriate. In order to limit the case load to numbers which could be managed by the available staff, random selection procedures were adopted for certain more common diagnoses in the West Midlands and North West. (All sarcomas, Wilms' tumours, germ cell tumours, lymphomas, hepatoblastomas and epithelial tumours were included; one-third of all other cases were eliminated).

Paediatric histopathologists and haematologists in each of the regions are members of a national paediatric oncology group, permitting confident classification of the children's tumours. Moreover, the majority of the children were entered into clinical trials, for many of which the diagnoses were also confirmed by review panels of paediatric pathologists. Inter-regional standardisation of diagnoses was thus achieved for the majority of cases. In Manchester all cases were reviewed by the Manchester Children's Tumour Registry Pathologists Panel.

*Selection of controls*

After the case child's parents had agreed to take part in the study two controls were selected for each case. The controls were matched for sex and age according to the following rules:

(a) for case children aged below two years the control child may be up to 3 months older but no younger;

(b) for case children aged between 2 and 10 years the control child may be up to 6 months older but no younger;

(c) for case children aged between 11 and 14 years the control child may be up to 6 months older or younger.

The first set, designated GP controls, was chosen using a random selection procedure from the lists of the general practitioners (GP) or group practices with whom the cases were registered, and the only exclusions were children who had suffered neoplastic disease. The second set was chosen from among children in hospital for reasons other than neoplastic disease and who did not have any of the following: (a) a genetic or other constitutional disease or malformation known to be associated with increased risk of cancer, e.g. Down's syndrome, hemihypertrophy, Beckwith-Wiedemann syndrome, aniridia, von Hippel-Lindau syndrome; (b) any other major malformation or chronic disease, e.g., major skeletal anomalies, neural tube defects, chromosomal disorders, Still's disease, epilepsy, diabetes or major blood disorders.

Children with minor malformations, e.g. squint, and less serious chronic conditions, e.g. hay fever, were considered eligible providing that the condition was not the reason for the child's admission to hospital. Controls were selected mainly from among acute surgical and accident cases. These children are designated 'hospital controls'.

In each region arrangements were made with appropriate local hospitals for selection of controls from lists of paediatric admissions and approach to potential control parents. The selection of hospital controls was carried out by the research associate.

Detailed instructions on GP control selection were sent to respective GPs who were given the option of selecting suitable controls themselves or allowing a member of the IRESCC staff to make the selection. The standard procedure recommended by the Royal College of General Practitioners was used. Where a GP made the selection himself those selected were checked for eligibility by IRESCC staff. Lists of all eligible hospital and GP controls for each case were compiled and their names arranged in a random order. After checking with the consultant or GP of the first child on the respective list that there was no objection, the parents were approached. The parents of second and subsequent children on the control lists were approached only if the parents of the first child were not willling to take part in the study. Each child selected as a control was equally well matched with the case.

*Approach to parents*

Wherever possible parents of cases and hospital controls were personally approached by the research associate. The study was explained to them, including the type of questions which would be asked at the interview, and written information about the study and the interview was also handed to them. Having gained their co-operation, an appointment for the interview was made. The great majority of case parents, but fewer hospital control parents, were contacted in this way. GP control parents and the parents of cases and hospital controls where a personal approach was not possible were contacted by letter, and similar written information concerning the study and the types of question which would be asked at interview were enclosed, together with a reply card. If no reply was received after two to three weeks, the parents were contacted again, by telephone if possible, and if the parents did not agree to interview at this stage then those cases were excluded from the study or alternative controls were sought as applicable. When a case child had died, the research associate was advised by the child's consultant or GP about a suitable time to approach the parents. Bereaved parents were approached only once, generally by letter, and if their agreement to participate in the study was not then secured the case was excluded.

*Interviews*

Interviews were usually carried out in the parents' homes but occasionally took place in hospital. The interviews were conducted according to a standard technique which had been agreed and piloted before the main study began. Group discussions with the research associates concerning interpretation and administration of the interview pro-forma were held at the outset of the study. Practice interviews with parents of ineligible cases were carried out and subsequently discussed. The research associates thus became familiar with the pro-forma and the agreed interview technique, before embarking on interviews with eligible case parents. The interview forms included questions on the index child's antenatal and previous medical history, congenital malformations, immunizations and exposures to X-rays, drugs and other potential carcinogens. Check-lists of illnesses, malformations and drugs were used in conjunction with the questionnaire to prompt more accurate recall of information. Details were also taken of the parents' past medical histories, occupations, exposures to potential carcinogens and mother's obstetric history. History of neoplasms, genetic disease and other chronic disorders was obtained for siblings and their offspring, grandparents, aunts, uncles and first cousins.

*Verification of information obtained at interview*

After the interview the mother's obstetric notes were obtained and details of the index pregnancy were abstracted onto a standard form. Information on the outcome of the mother's other pregnancies and any gynaecological problems was also recorded. The GP's records were abstracted for details of the health of the mother and child, particularly the health of the mother during the index pregnancy.

If the index child had been admitted to hospital for any serious condition the hospital notes were obtained and details were similarly abstracted. Many illnesses in siblings, parents and other relatives of the index child reported at interview were confirmed by reference to hospital or other medical records and standard abstract forms were completed. Priority was given to the verification of the following diagnoses in the index children and their relatives: neoplastic disease, congenital malformations, any known genetic disease and certain major chronic conditions, e.g. diabetes and rheumatoid arthritis. Hospital notes were used as the main source for verification, but if these were not available other sources such as cancer registrations and death certificates were used. Thus for each case entered into the study a completed set of data comprises interview pro-formas for the case and two matched controls, and abstract forms completed from medical records with respect to the mothers' obstetric histories and medical histories of the index children and their relatives. A completed data set is hereafter referred to as a 'triplet'.

*Preparation of data for analysis*

ICD-0 and ICD 9 (WHO 1976, 1978) were used to code neoplastic and other diseases respectively. Code lists were devised for the remaining data such that the information could be coded in detail. Each region coded approximately one-third of the interview form and the respective medical abstract form derived from all three regions. Thus the same portion of the data provided from each of the three separate centres was coded by one centre. Information from the interview form was combined with but coded separately from information on the medical abstract form. Identifying data relating to the index child was coded in a purely numerical form to preserve total confidentiality. The data were subsequently pooled for analysis.

### Results

During the study period there were 761 incident cases of childhood neoplasia in the three regions. One hundred and forty six of these were excluded mainly as a result of the procedures in operation in the West Midlands and the North West to limit the case load. Therefore, 615 cases were considered eligible for interview and 555 interviews were successfully obtained – 210 in the West Midlands, 182 in the North West and 163 in Yorkshire. Of the 615 eligible cases 19 sets of parents were not approached because either their GP or their consultant thought that they would find it too distressing to take part in the study. Of the 596 sets of parents approached 41 did not wish to take part in the study, i.e. 93% of case parents approached agreed to be interviewed. Refusal rate among case parents was similar in the three regions.

The distribution of diagnoses among interviewed, non-interviewed and excluded cases is shown in Table I. Forty-four per cent of interviewed cases had leukaemias and other reticuloendothelial neoplasms. Fifteen per cent and 13% had central nervous system and connective tissue tumours respectively. Wilms' tumours and neuroblastomas each comprised ~6% and just over 7% had germ cell and trophoblastic tumours. Other rare tumours represented in the study included retinoblastoma, hepatoblastoma and various carcinomas.

Interviews with parents of GP and hospital controls fulfilling the defined matching criteria were carried out for each of the cases. Over 72% of GP controls were ranked first and 20% second on the

**Table I** Distribution by diagnosis of interviewed and non-interviewed cases among all eligible cases

| Diagnosis | Interviewed cases n | (%) | Number NOT interviewed: Parent refusal n | GP/consultant refusal n | Exclusion n |
|---|---|---|---|---|---|
| Leukaemia | 171 | (30.8) | 12 | 4 | 45 |
| Lymphoma and other reticulo-endothelial neoplasms | 74 | (13.3) | 1 | 2 | 7 |
| Central nervous system | 81 | (14.6) | 14 | 5 | 57 |
| Soft tissue sarcoma | 42 | (7.6) | 1 | 0 | 3 |
| Bone tumours | 30 | (5.4) | 1 | 2 | 9 |
| Wilms' and other complex renal tumours | 35 | (6.3) | 1 | 0 | 1 |
| Neuroblastoma | 35 | (6.3) | 2 | 1 | 11 |
| Retinoblastoma | 6 | (1.1) | 1 | 0 | 2 |
| Hepatoblastoma | 6 | (1.1) | 1 | 0 | 0 |
| Germ cell and trophoblastic tumours | 41 | (7.4) | 2 | 2 | 3 |
| Malignant epithelial tumours | 20 | (3.6) | 3 | 0 | 2 |
| Other malignant tumours | 3 | (0.5) | 0 | 0 | 1 |
| Other benign tumours | 11 | (2.0) | 2 | 3 | 5 |
| Total | 555 | (100.0) | 41 | 19 | 146 |

lists of eligible controls. Among the hospital controls 64% were ranked first and 22% second on their respective lists. There was little variation between regions with respect to rank order of controls interviewed, except that a higher proportion of hospital controls from Yorkshire were ranked third or higher, i.e. 28% Yorkshire compared with 11% Birmingham and 6% Manchester.

Mothers of cases and controls were considered to be the main source of information, but we aimed to have both mother and father present at the interview whenever possible. This was achieved for ~59% of cases and 50% of each set of controls. The majority of the remaining interviews were carried out with the mother alone, but occasionally another relative, usually the maternal grandmother, was also present at the interview (Table II). Interviews were carried out at a place convenient to the parents, and for controls 98% were carried out in homes, but 39% of case interviews were carried out in the hospital. This was often necessary because parents, especially mothers, would be spending most of their time at the hospital with their sick child.

**Table II** Present at interview

| | Mother and father n | (%) | Mother only n | (%) | Mother and other relative n | (%) |
|---|---|---|---|---|---|---|
| Case | 326 | (58.7) | 203 | (36.6) | 26 | (4.7) |
| GP control | 278 | (50.1) | 260 | (46.8) | 17 | (3.1) |
| Hospital control | 276 | (49.7) | 266 | (47.9) | 13 | (2.3) |

Mean interview length was 53 min in Yorkshire (55 min for cases, 51 min for GP controls and 53 min for hospital controls); 96 min in the West Midlands (114 min for cases, 83 min for GP controls and 90 min for hospital controls); and 69 min in Manchester (74 min for cases and 66 min for both GP and hospital controls). The relatively longer interview times in the West Midlands can be partly explained due to extended pedigree information being gathered in this region. It was thought that variations in interview length may also be related to ethnic group and this was examined (Table III). There were 110 families in the study population who were specified as belonging to an ethnic group other than 'White European'. Interview lengths did not markedly differ between ethnic groups and no relation was found between interview length and level of education of the parents.

Obstetric records were routinely sought and abstracted after each interview and Table IV shows the success of this exercise. On average 88% of sets of obstetric notes were abstracted and the degree of success was remarkably similar between regions and

**Table III** Interview length (min)

| | <40 | | 40–59 | | 60–79 | | 80+ | | NR | | Total | |
|---|---|---|---|---|---|---|---|---|---|---|---|---|
| | *n* | (%) | *n* | (%) | *n* | (%) | *n* | (%) | *n* | (%) | *n* | (%) |
| White European | 148 | (9.6) | 564 | (36.6) | 248 | (16.1) | 513 | (33.2) | 69 | (4.5) | 1542 | (92.6) |
| Indian/Pakistani | 6 | (7.4) | 24 | (29.6) | 14 | (17.3) | 31 | (38.3) | 6 | (7.4) | 81 | (4.9) |
| West Indian | 4 | (15.4) | 7 | (26.9) | 2 | (7.7) | 11 | (42.3) | 2 | (7.7) | 26 | (1.6) |
| Other and unspecified | 1 | (6.3) | 7 | (43.7) | 3 | (18.7) | 5 | (31.3) | 0 | | 16 | (0.9) |
| Total | 159 | | 602 | | 267 | | 560 | | 77 | | 1665 | (100) |

NR = not recorded.

**Table IV** Availability of obstetric records to the study

| | Obstetric notes abstracted | | Obstetric notes not available: Destroyed | | Home delivery | | Other reason | |
|---|---|---|---|---|---|---|---|---|
| | *n* | (%) | *n* | (%) | *n* | (%) | *n* | (%) |
| Case | 489 | (88) | 24 | (4) | 26 | (5) | 16 | (3) |
| GP control | 493 | (89) | 24 | (4) | 22 | (4) | 16 | (3) |
| Hospital control | 487 | (88) | 14 | (2) | 32 | (6) | 22 | (4) |

**Table V** Availability of mother's GP records to the study

| | GP records abstracted | | GP records not abstracted | |
|---|---|---|---|---|
| | *n* | (%) | *n* | (%) |
| Case | 506 | (91) | 49 | (9) |
| GP control | 517 | (93) | 38 | (7) |
| Hospital control | 514 | (93) | 41 | (7) |

**Table VI** Availability of other medical records to the study

| | Number of records sought | Number of records abstracted | (%) | Average number of records abstracted per family |
|---|---|---|---|---|
| Case | 1552 | 1237 | (80) | 2.2 |
| GP control | 1165 | 889 | (76) | 1.6 |
| Hospital control | 1349 | 1062 | (79) | 1.9 |

between cases and the two sets of controls within each region. Failure to abstract the relevant obstetric records was usually because of unavailability of the notes. Project staff were very rarely denied access to the records. A similar uniformly high level of success was achieved with respect to obtaining access to GP records (Table V).

Table VI shows the numbers of other types of medical records from which information was abstracted. There was less uniformity between regions and between cases and controls with respect to this information, but more flexibility was allowed with respect to which reported diagnoses would be verified.

### Discussion

Successful case-control studies, especially for rare cancers, are best derived from large populations of diagnostically confirmed cases, i.e. from good cancer registries (Frentzel-Beyme & Wagner, 1979). However, cancer registration is not universally complete, and histopathological classification of childhood cancers in particular is difficult. Two of the strengths of the IRESCC study were the known high ascertainment of childhood cancer cases in the three regions, and the reliability of the histopathological diagnoses. Centralised paediatric oncology services were in operation, and although case ascertainment was population-based, the majority of patients were treated in the oncology centres.

The distribution of diagnoses amongst cases interviewed is very similar to that seen among the childhood population of Great Britain as a whole (Draper *et al.*, 1982). The exceptions were tumours of the central nervous system, which were comparatively under-represented. However, the sample of 81 central nervous system tumours included in the study comprised examples of each of the main types of brain tumour seen in children (e.g. astrocytomas, medulloblastomas and ependymomas) in addition to rarer types of intracranial tumour (e.g. craniopharyngioma and less common types of glioma). Relatively fewer central nervous system tumours were treated in the paediatric oncology centres compared with other tumours, and this probably accounts for the lower success rate in obtaining interviews with the parents of these cases. Nevertheless the study covers a representative sample of childhood malignant disease.

A very high proportion of parents approached to take part in this study agreed to participate. The research associates worked in close liaison with the medical staff responsible for the care of the children. Whenever possible, the approach to the parents was made at a point of optimism in the child's treatment, usually when the child had completed his initial therapy and was well. At the time of interview 96% of the case children were alive, in contrast to many other studies e.g. The Oxford Survey of Childhood Cancer, where the majority, if not all, interviews were carried out a considerable time after the child's death (Stewart & Kneale, 1970; Sanders & Draper, 1979; Swerdlow *et al.*, 1982). Our type of approach is recommended for any future interview studies of the aetiology of childhood cancer.

Selection of controls is of paramount importance in any case-control study. Poor selection can lead to biases and spurious results. The design of our study involved selection of two controls per case from two sources. The GP controls represent random neighbourhood controls, and hospital controls were selected in part to overcome possible lack of recall by the GP control parents, since the hospital control parents had experienced the child's admission to hospital and the taking of a medical history.

Reasons for this type of design were: firstly, to add power to the analyses of results, and secondly, to avoid some of the biases which may accumulate in a control group selected from only one source, even though more than one control may be used (Cole, 1979). Most previous case-control studies of childhood cancer have used one control per case. Two were used in studies of brain tumours (Gold *et al.*, 1979); parental occupation (Kwa & Fine, 1980; Hemminki *et al.*, 1981); and tonsillectomy and Hodgkin's Disease (Vianna *et al.*, 1980). Three controls were used in a study of environmental factors in the aetiology of 33 cases of rhabdomyosarcoma (Grufferman *et al.*, 1982). The sources of controls in other reported studies of childhood cancer have included birth registers (e.g. Stewart & Kneale, 1970; Bithell *et al.*, 1973; Kwa & Fine, 1980; Grufferman *et al.*, 1982); population registers (e.g. Abramson *et al.*, 1978; van Steensel-Moll *et al.*, 1983); or tax lists (Dean *et al.*, 1973). Siblings were used as controls by Vianna *et al.* (1980), and children with cancers of different types from those being studied, by Gold *et al.* (1979) and Swerdlow *et al.* (1982).

In our study, although each of the several hospital and GP controls selected for each case was equally well matched with that case, some biases may have been operating with respect to which set of parents agreed to be interviewed, for example, parents from higher socio-economic groups may have been more willing to take part in the study. Similarly, parents who were not native English speakers may have been reluctant to do so because of language difficulties, even though interpreters were offered in these circumstances. However, since more than 90% of GP controls and more than 85% of hospital controls were ranked first or second on the control selection lists, we believe that this should not lead to biases in the controls.

The policy was to interview both parents, and this was achieved for half the controls and more than half of the cases. To have included the father in a higher proportion of the interviews would have meant conducting even more during evenings and weekends. The purpose of having the father present at the interview in addition to the mother was so that he could give more detail about his own and his family's medical histories, and the fact that proportionately more fathers of cases were present at the interview than fathers of controls will be taken into account when analysing certain sections of the data. Since the parents were given written information about the types of questions which would be asked well before the interview, the absence of the father need not necessarily result in any serious deficiencies in the data.

The interview concentrated on factors associated with the child's pre-natal life i.e., drugs and other substances which may cross the placenta; obstetric investigative procedures which may affect the foetus; and genetic factors. More emphasis has been placed on pre-natal factors in the present study than in previous case-control studies of childhood cancer (e.g. Sanders & Draper, 1979; Grufferman *et al.*, 1982), and much more detail has been collected on, for example, pregnancy drugs, and more extended pedigree information, than in these previous studies. Conversely, perhaps less emphasis has been placed on post-natal events.

Recall of the interviewed subject may be greater for cases than controls, because the occurrence of disease in the cases tends to prompt recall (Feinstein, 1979). The control group should therefore be stimulated to achieve similar recall, and efforts should be (but are rarely) made to validate statements given at interview. In the IRESCC study, parents of both cases and controls were given written and sometimes verbal information about the study, which we hoped would prompt family discussions and recall. We also undertook comprehensive validation of information reported at interview by checking hospital records, death certificates and cancer registry records. The extent to which verification of information gained at interview by reference to medical records was carried out distinguishes IRESCC from other studies. This exercise proved highly successful, and is a reflection of the degree of interest in the study shown by medical personnel in all three participating regions. The effects of such verification of interview information on the eventual results of case-control studies is discussed in detail elsewhere (McKinney *et al.*, in preparation).

Joint discussion sessions with the research associates from each region were held to ensure uniformity of interview technique. Nevertheless there was variation between the regions with respect to length of interview, but since the data will be analysed using matched case-control comparisons, differences between regions in the exact way in which the interview was carried out are not felt to be important.

Childhood cancer is rare, and a general practitioner could expect to see only a very few cases in his working lifetime. Perhaps because of this, the GPs of the children included in the study were very helpful. Although priority was given to the verification of certain diagnoses in relatives of the index child, there were guidelines rather than rigid rules for this aspect of the study. Also, in the West Midlands, consultants were asked to complete questionnaires concerning illnesses among other family members rather than to lend the notes. This accounts for the larger number of records sought in Birmingham, since this was a less time-consuming procedure than that adopted in Leeds and Manchester. These points will be taken into consideration when interpreting results of analyses of these data.

The fact that the study was being carried out in close collaboration with the consultants responsible for the treatment of the children may also account for the extremely co-operative attitude shown by all those approached to assist with the study.

For the purposes of coding the complete data set consisting of interview pro formas for the case and two matched controls, and abstract forms with respect to obstetric, GP and other medical records, the method of apportioning the data between the three regions allowed expertise to be gained in a limited range of codes. This ensured greater consistency and accuracy than would have been inherent in a system where each centre coded all its own data. The coding of information abstracted from medical records alongside but distinct from the interview information will allow the two sets of information to be dealt with separately if this is desirable for particular analyses. The total data set consists of approximately one million separately coded items of information.

In conclusion, IRESCC is an aetiological study of childhood cancer with a number of unusual features. The data collection exercise has been extremely successful, and we anticipate that careful and detailed analyses of the data will yield interesting and informative results.

We thank the parents of the children included in the study and the many general practitioners, consultants and nurses in the three regions who assisted us. Also, the Cancer Research Campaign, the Leukaemia Research Fund, the Department of Health and Social Services, the Scottish Home and Health Department, the Special Trustees for the former United Birmingham Hospitals Trust Funds and the Special Trustees of Leeds Western Health Authority for financial support. We thank Dr H.G. Frank, Dr E. Hill and many other paediatricians, surgeons and radiotherapists whose patients were included; the medical records officers and Cancer Registries in the three regions for their help. We thank Dr D.I.K. Evans, Dr F.G.H. Hill and Dr M.F. Greaves for haematological confirmation of diagnoses; Office of Population Censuses and Surveys for access to death certificates; Mrs P. Brown, Mr R.W. Boyko, Mrs C. Christmas, Mrs P. Dilworth, Miss C. Kite, Miss P.M. Landells, Mrs A. Mainwaring, Miss G. Mason, Mrs J. Olden, Mrs E.M. Roberts, Dr M. Potok, Mrs S. Warner and Mr D. Winterburn for secretarial, statistical and computing assistance; Rank Xerox for photocopying; Bell & Howell for the use of a microfilming camera; Systime Ltd. for the gift of a computer and the University of Leeds for the use of the Amdahl computer.

### References

ABRAMSON, H., PRIDAN, H., SACKS, M.I., AIVTZOUR, M. & PERITZ, E. (1978). A case-control study of Hodgkin's disease in Israel. *J. Natl Cancer Inst.*, **61**, 307.

BIRCH, J.M. (1983). Epidemiology of paediatric cancer. In *Recent Results in Cancer Research: Paediatric Oncology*, Duncan, W. (ed) Chap. 1, p. 1. Springer-Verlag: Berlin.

BITHELL, J.F., DRAPER, G.J. & GORBACH, P.D. (1973). Association between malignant disease in children and maternal virus infections. *Br. Med. J.*, **1**, 706.

COLE, P. (1979). The evolving case-control study. In *The Case Control Study. Consensus and Controversy*, Ibrahim, M.A. (ed) p. 15. Pergamon Press: New York.

DEAN, A.G., WILLIAMS, E.H., ATTOBUS, G., OMEDA, J., GADI, A., AMUTI, A. & ATIMA, S.B. (1973). Clinical events suggesting herpes simplex infection before onset of Burkitt's lymphoma. *Lancet*, **ii**, 1225.

DRAPER, G.J., BIRCH, J.M., BITHELL, J.F. & 6 others. (1982). Childhood cancer in Britain: Incidence, survival and mortality. (Studies on Medical and Population Subjects No. 37). HMSO for OPCS: London.

FEINSTEIN, A.R. (1979). Methodological problems and standards in case-control research. In *The Case-Control Study. Consensus and Controversy*, Ibrahim, M.A. (ed) p. 35. Pergamon Press: New York.

FRANTZEL-BEYME, R.R. & WAGNER, G. (1979). The importance of epidemiology in the study of causes of bone tumours. *Path. Res. Pract.*, **166**, 31.

GOLD, E., GORDIS, L., TONASCIA, J. & SZKLO, M. (1979). Risk factors for brain tumours in children. *Amer. J. Epidem.*, **109**, 309.

GRUFFERMAN, S., WANG, H.H., DELONG, E.R., KIMM, S.Y.S., DELZELL, E.S. & FULLETTA, J.M. (1982). Environmental factors in the aetiology of rhabdomyosarcoma in childhood. *J. Natl Cancer Inst.*, **68**, 107.

HEMMINKI, K., SALONIEMI, I., SALONEN, T., PARTANEN, T. & VAINIO, H. (1981). Childhood cancer and parental occupation in Finland. *J. Epidem. Comm. Health*, **35**, 11.

KWA, S.-L. & FINE, L.J. (1980). The association between parental occupation and childhood malignancy. *J. Occ. Med.*, **22**, 792.

LECK, I., BIRCH, J.M., MARSDEN, H.B. & STEWARD, J.K. (1976). Methods of classifying and ascertaining children's tumours. *Br. J. Cancer*, **34**, 69.

OFFICE OF POPULATION CENSUSES AND SURVEYS (1982). Mortality Statistics Series DH 2 No. 7 for 1980. HMSO: London.

SANDERS, B.M. & DRAPER, G.J. (1979). Childhood cancer and drugs in pregnancy. *Br. Med. J.*, **1**, 717.

STEWART, A. & KNEALE, G.W. (1970). Radiation dose effects in relation to obstetric X-rays and childhood cancers. *Lancet*, **i**, 1185.

SWERDLOW, A.J., STILLER, C.A. & KINNIER WILSON, L.M. (1982). Pre-natal factors in the aetiology of testicular cancer: an epidemiological study of childhood testicular cancer deaths in Great Britain, 1953–73. *J. Epidem. Comm. Health*, **36**, 96.

VAN STEENSEL-MOLL, H.A., VALKENBURG, H.A. & VAN ZANEN, G.E. (1985). Childhood leukemia and parental occupation. A register-based case-control study. *Am. J. Epidem.*, **121**, 216.

VIANNA, N.J., LAWRENCE, C.E., DAVIES, J.N.P., ARBUCKLE, J., HARRIS, S., MARANI, W. & WILKINSON, J. (1980). Tonsillectomy and childhood Hodgkin's disease. *Lancet*, **ii**, 338.

WATERHOUSE, J.A.H. (1982). Cancer incidence in West Midlands 1973–77. In *Cancer Incidence in 5 Continents*, Waterhouse *et al.* (eds) Vol. IV, 550 (IARC Scientific Publication No. **42**), Lyon.

WORLD HEALTH ORGANISATION (1976). International Classification of Diseases for Oncology. ICD-O. WHO: Geneva.

WORLD HEALTH ORGANIZATION (1978). International Classification of Diseases. Manual of the International Statistical Classification of Diseases, Injuries, and Causes of Death. Ninth Revision. HMSO: London, by arrangement with the WHO, Geneva.